# Oxaliplatin-induced blood brain barrier loosening: a new point of view on chemotherapy-induced neurotoxicity

**DOI:** 10.18632/oncotarget.25193

**Published:** 2018-05-04

**Authors:** Jacopo Junio Valerio Branca, Mario Maresca, Gabriele Morucci, Matteo Becatti, Ferdinando Paternostro, Massimo Gulisano, Carla Ghelardini, Daniela Salvemini, Lorenzo Di Cesare Mannelli, Alessandra Pacini

**Affiliations:** ^1^ Department of Experimental and Clinical Medicine, Anatomy and Histology Section, University of Florence, Florence, Italy; ^2^ Department of Neuroscience, Psychology, Drug Research and Child Health (NEUROFARBA), Pharmacology and Toxicology Section, University of Florence, Florence, Italy; ^3^ Department of Experimental and Clinical Biomedical Sciences “Mario Serio”, University of Florence, Florence, Italy; ^4^ Department of Pharmacology and Physiology Saint Louis University, Saint Louis, Missouri, United States

**Keywords:** blood brain barrier, RBE4 cell line, Oxaliplatin, tight junctions, neuropathic pain

## Abstract

Oxaliplatin is a key drug in the treatment of advanced metastatic colorectal cancer. Despite its beneficial effects in tumor reduction, the most prevalent side-effect of oxaliplatin treatment is a chemotherapy-induced neuropathy that frequently forces to discontinue the therapy. Indeed, along with direct damage to peripheral nerves, the chemotherapy-related neurotoxicity involves also the central nervous system (CNS) as demonstrated by pain chronicity and cognitive impairment (also known as chemobrain), a newly described pharmacological side effect.

The presence of the blood brain barrier (BBB) is instrumental in preventing the entry of the drug into the CNS; here we tested the hypothesis that oxaliplatin might enter the endothelial cells of the BBB vessels and trigger a signaling pathway that induce the disassembly of the tight junctions, the critical components of the BBB integrity.

By using a rat brain endothelial cell line (RBE4) we investigated the signaling pathway that ensued the entry of oxaliplatin within the cell. We found that the administration of 10 μM oxaliplatin for 8 and 16 h induced alterations of the tight junction (TJs) proteins zonula occludens-1 (ZO-1) and of F-actin, thus highlighting BBB alteration. Furthermore, we reported that intracellular oxaliplatin rapidly induced increased levels of reactive oxygen species and endoplasmic reticulum stress, assessed by the evaluation of glucose-regulated protein GRP78 expression levels. These events were accompanied by activation of caspase-3 that led to extracellular ATP release.

These findings suggested a possible novel mechanism of action for oxaliplatin toxicity that could explain, at least in part, the chemotherapy-related central effects.

## INTRODUCTION

Chemotherapy-induced neuropathy (CIN) is a frequent side effect for many of the most common cancer treatments and is predominantly characterized by sensory symptoms like pain, paresthesia and dysesthesia; motor and autonomic symptoms can develop dependently by the drug. CIN can be disabling, causing significant loss of functional abilities and decreasing quality of life [1]. The occurrence of CIN has been studied for decades and it is commonly associated with well-known anticancer drugs administration including oxaliplatin [2, 3]. CIN is thought to be caused by drug-induced damage to components of the peripheral nervous system (PNS) and this structural damage results in abnormal somatosensory processing in the central nervous system (CNS) [4]. On the other hand, increasing evidence suggest a direct, neurotoxic effect of chemotherapeutics to the CNS. A remarkable reorganization of spinal and supraspinal areas after anticancer treatments was highlighted [5, 6]. Accordingly, our group has demonstrated that during oxaliplatin-dependent neuropathic pain a significant increase in glial cells (microglia and astrocytes) [7, 8] as well as molecular, electrophysiological and redox alterations has been evidenced in spinal cord and many brain areas [9–11]. Furthermore, many studies have found evidence supporting the influence of chemotherapy on cognitive functioning [12–14], describing a new neuropsychological syndrome associated with cancer therapy named chemotherapy-induced cognitive impairment (CICI) or chemobrain characterized by memory, learning and motor function impairment [15–18].

Although many authors have demonstrated that the presence of the blood-brain barrier (BBB) limits access of many anticancer drugs to the brain and that the cerebrospinal fluid (CSF) penetration of the oxaliplatin is limited [19] a direct action of oxaliplatin on BBB endothelial cells (EC), the core element of BBB, could not be ruled out.

EC have been demonstrated to play a key role in BBB properties as they form the anatomic basis of the barrier [20, 21]. These cells differ fundamentally from other vascular endothelia in their capacity to regulate the passage of molecules and cells to and from the neural parenchyma [22]. The capillary endothelium in the brain is tighter than peripheral microvessels, the cytoplasm has an uniform thickness with no *fenestrae*, and a continuous basement membrane [23, 24]. Most importantly, EC lining the vascular wall have narrow junctional complexes that includes mainly tight junction (TJ) and adherens junction (AJ) proteins [25, 26]. They eliminate gaps or spaces between cells and prevent any free diffusion of blood-borne substances into the brain parenchymal space [27]. Neurotoxics may evoke strong or subtler mechanisms leading to BBB disruption or dysfunction. Increased cerebrovascular permeability can be implemented via the paracellular or the transcellular route, or both [28]. Several molecular mechanisms participate to the dysregulation of interendothelial junctions: inflammatory factors such as TNF-α, IL-1β, IL-6, or free radicals, or bradykinin, or angiogenic factors such as VEGF [29–31]. These factors, strongly related to chemotherapy-induced neurotoxicity, can mediate a downregulation of junctional proteins as well as subcellular redistribution, cytoskeletal rearrangements and a direct disruption of interendothelial junctions. Thus, it was our purpose to identify oxaliplatin cytotoxic profile and the molecular pathway related to oxaliplatin-dependent BBB alteration in an immortalized rat brain endothelial cell line (RBE4 cells) that, retaining a stable phenotype of BBB endothelium *in vivo*, are a ductile and widely accepted *in vitro* model for the study of the BBB [32].

## RESULTS

### MTT assay

**Figure 1:**
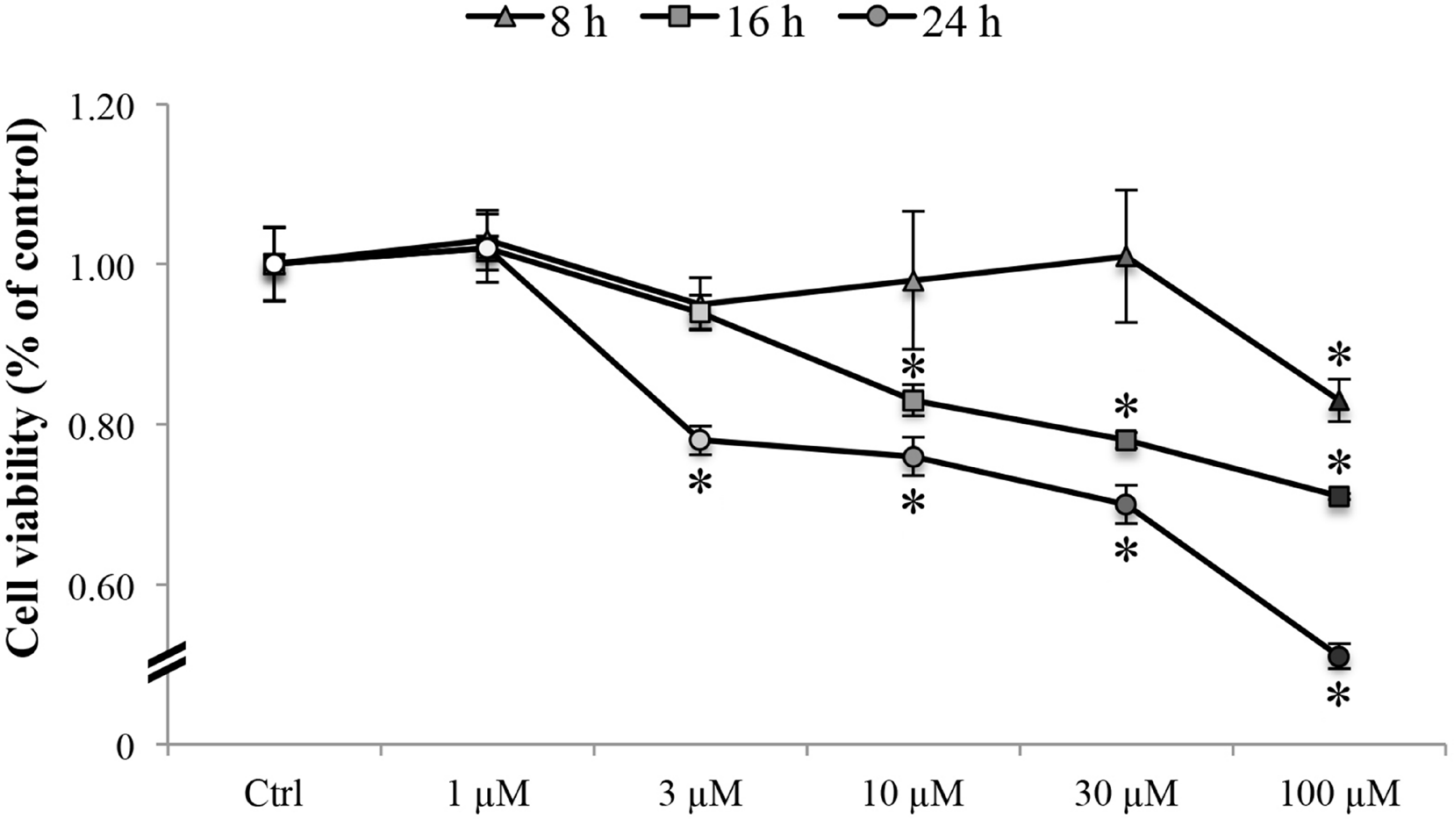
RBE4 cell viability RBE4 cells (2.5 × 10^4^ cells/well) were incubated with oxaliplatin (1–100 μM) for 8, 16 or 24 h. Viability was quantified by MTT assay; absorbance was measured at 570 nm. Values are expressed in percentage of control absorbance as the mean ± S.E.M. of five independent experiments. Control condition absorbance was fixed at 100%. ^*^p < 0.05 vs control.

Results from the cytotoxicity assays are represented in Figure 1. A concentration range of oxaliplatin from 1 to 100 μM was tested at different time points (8, 16 and 24 hours). Results show that oxaliplatin was able to induce a decrease in cell viability in a dose- and time-dependent manner. The drug starts to alter the metabolic activity of RBE4 cells after 8 h (Figure 1, triangles) of treatment. The lowest tested concentration (1 μM) did not alter cell viability at any time point, whereas an oxaliplatin concentration of 3 μM induced a significant (^*^p < 0.05 vs control) decrease in cell viability only at 24 h (Figure 1, circles). On the contrary, at all time points significant (^*^p < 0.05 vs control) effects on RBE4 cells were observed at the highest concentration (100 μM). In the concentration range between 3 μM and 100 μM, cells exhibited a significant (^*^p < 0.05 vs control) and strong decrease in cell viability at 16 (Figure 1, squares) and 24 hours (Figure 1, circles) after oxaliplatin treatment.

Based on previously reported data concerning patients’ plasma concentration after oxaliplatin repeated treatments [35–37], we adopted the best experimental conditions that could better reflect an *in vivo* situation, mimicking chronic neuropathy. For this reason, we chose the concentration of 10 μM that induced a low level of mortality but, at the same time, enabled us to study the oxaliplatin effects at the cellular level.

**Figure 2:**
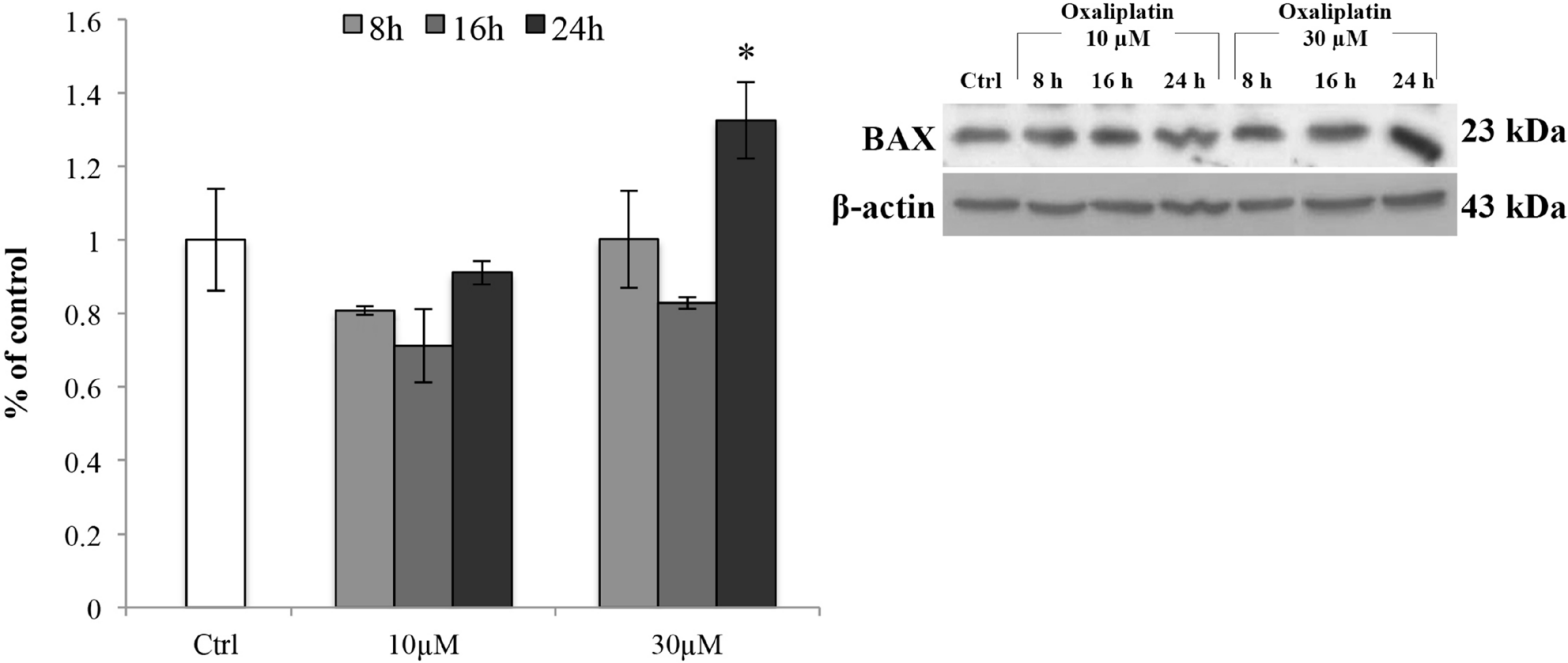
BAX protein expression levels Representative Western blot of the effects of oxaliplatin (10 and 30 μM) on the protein levels of BAX after 8, 16 and 24 h of treatment. Bars represent the mean ± S.E.M., *n* = 4; ^*^p < 0.05 vs control (untreated cells).

Moreover, to ensure that the concentration of oxaliplatin 10 μM did not trigger the apoptotic pathway, we tested the expression levels of the pro-apoptotic protein BAX (Figure 2). The results showed that there was a significant (^*^p < 0.05 vs control) increase in the protein levels only at the concentration of 30 μM and after 24 h of exposure to the chemotherapeutic agent.

### Immunocytochemistry for ZO-1 and F-actin proteins

**Figure 3:**
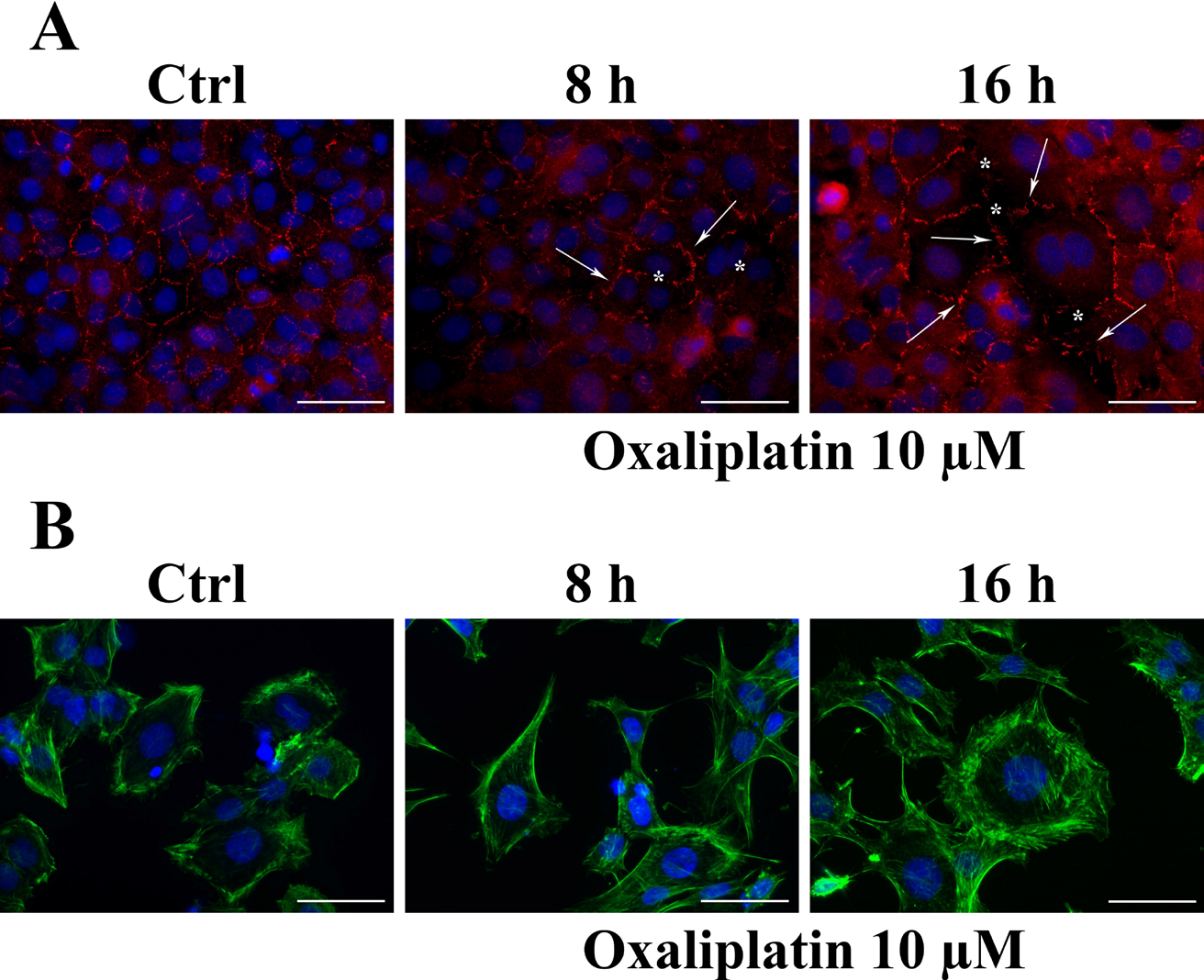
Effect of oxaliplatin on immunostaining for ZO-1 junctional protein and F-actin in RBE4 cells In (**A**) asterisks show holes formed between endothelial cells. Arrows indicate fragmentation, indicative for loss of junctional immunostaining. Figure (**B**) shows the oxaliplatin-dependent appearance of numerous stress fibers. Original magnification 400×. Bar: 50 μm. Each experimental point was performed in triplicate, for three different set of experiments. Pictures are representative of fifteen field captured for each experimental point.

The results related to BAX protein expression levels, prompted us to determine the effects of 10 μM of oxaliplatin on the expression and localization of ZO-1 and F-actin proteins on RBE4 cells at 8 and 16 hours post-treatment using immunohistochemistry. We found that the TJ protein ZO-1 localized to the cell-cell junctions, showing a more prominent and clear immunostaining at the cell contacts (Figure 3A, control), as well as F-actin exhibited a typical, marginal pattern of localization (Figure 3B, control). Eight hours of oxaliplatin treatment caused monolayer disruption, loss of ZO-1 staining, leading to a “zipper-like” staining pattern (Figure 3A, oxaliplatin) and holes that became visible between RBE4 cells, as well as the formation of numerous F-actin stress fibers instead of marginal localization (Figure 3B, oxaliplatin). These alterations were even more evident after 16 hours of treatment.

### Oxaliplatin-induced overproduction of intracellular ROS

**Figure 4:**
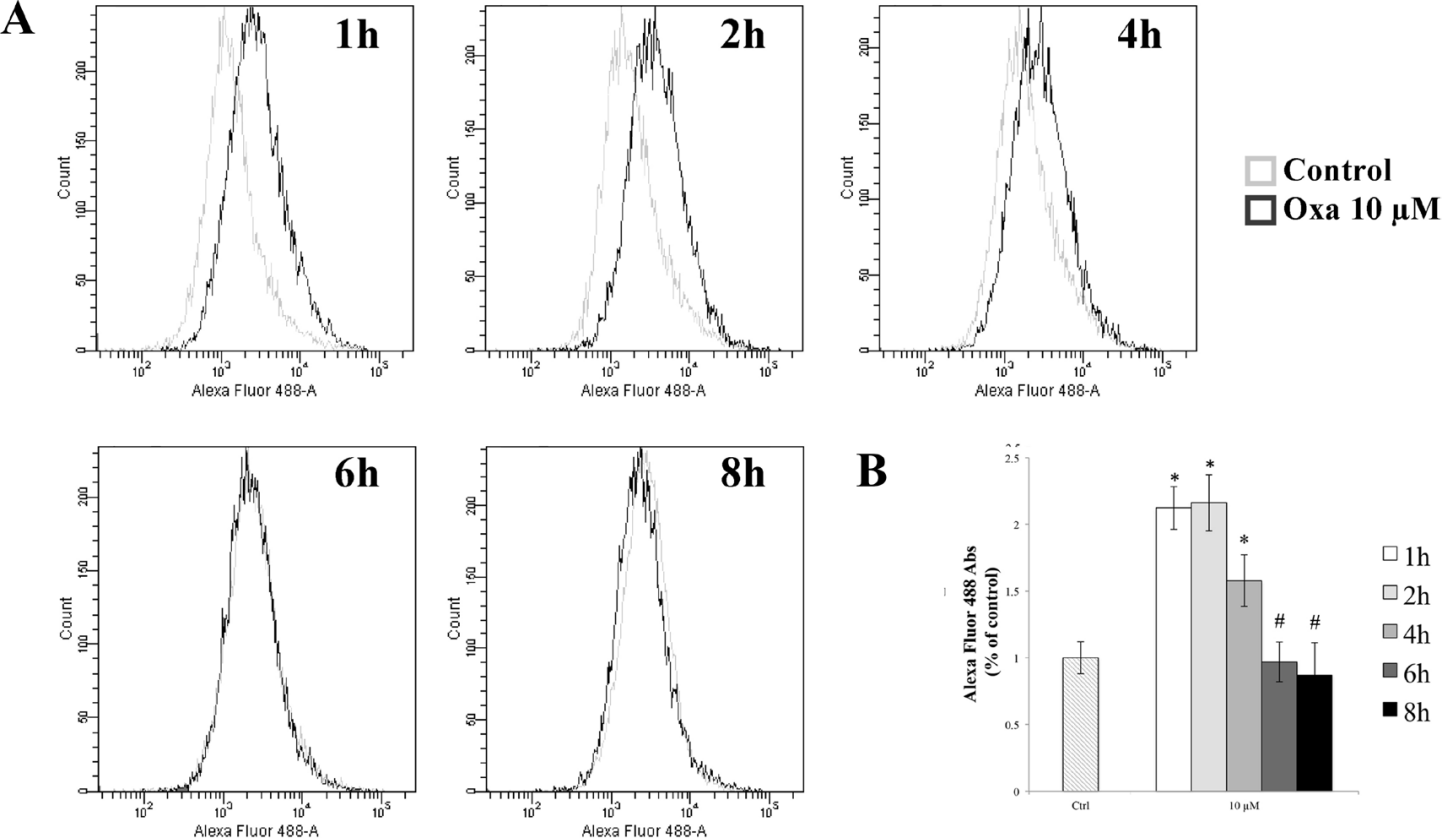
Evaluation of ROS generation (**A**) Time-course of ROS production by H_2_DCFDA fluorescence in control and in 10 μM oxaliplatin-treated RBE4 cells by FACS analysis. (**B**) The reported values (mean ± S.E.M.) are representative of five independent experiments, each performed in triplicate; ^*^p < 0.05 vs control; ^#^*p* < 0.05 vs 1 h.

In order to evaluate the role of oxidative stress in TJ alterations, we measured the intracellular ROS in RBE4 cells at different time points after 10 μM of oxaliplatin using the redox-sensitive fluoroprobe dye CM-H_2_DCFDA by FACS (fluorescence-activated cell sorting) analysis, as previously reported [38]. As shown in Figure 4, an increase in ROS production was seen following incubation of cells with 10 μM oxaliplatin for 1 and 2 hours, which remain significantly increased at 4 hours compared to control cells (^*^p < 0.05 vs control). The ROS levels decreased quickly, and then returned to normalcy after 6 h of treatment (^#^*p <* 0.05 vs 1 h), indicating that the increase of ROS generation was rapid and transient.

### Oxaliplatin-induced endoplasmic reticulum (ER) stress in RBE4 cells

**Figure 5:**
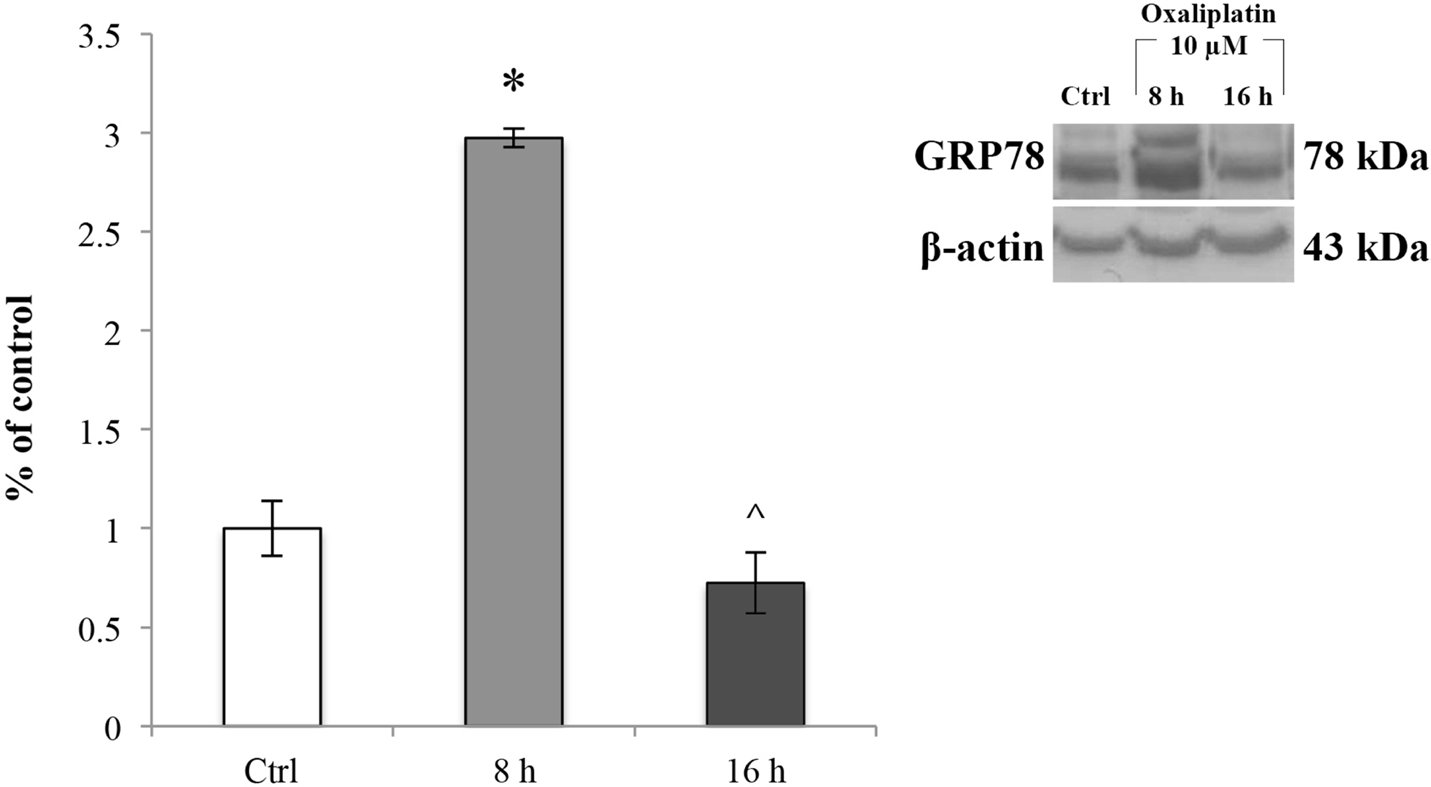
GRP78 expression levels in RBE4 cells treated with oxaliplatin Immunoblot analysis at 8 and 16 h exposure to 10 μM oxaliplatin and quantification of GRP78 expression at 8 and 16 h. Control condition was arbitrarily set as 100% and results are expressed as mean ± S.E.M. Results are representative of at least three independent immunoblots. Significant at ^*^p < 0.05 vs control and ^*p* < 0.05 vs 8 h.

We then assessed whether the ROS increase induced ER stress. To this end, we evaluated GRP78 protein expression levels in RBE4 cells treated with 10 μM oxaliplatin for 8 and 16 h by immunoblot analysis on total cellular homogenates. Values were normalized to β-actin protein expression. As shown in Figure 5, 10 μM oxaliplatin significantly (^*^p < 0.05 vs control) up-regulated GRP78 about three times compared to the control cells (Figure 5, light grey bar) at 8 h after the chemotherapy administration. This effect was time-dependent because the protein expression decreased (Figure 5, dark grey bar) to values comparable to that of control cells at 16 h of oxaliplatin treatment (^*p <* 0.05 vs 8 h).

Taken together, these data indicated that after oxaliplatin treatment RBE4 cells underwent ER stress.

### Oxaliplatin treatment elicits caspase-3 activation

**Figure 6:**
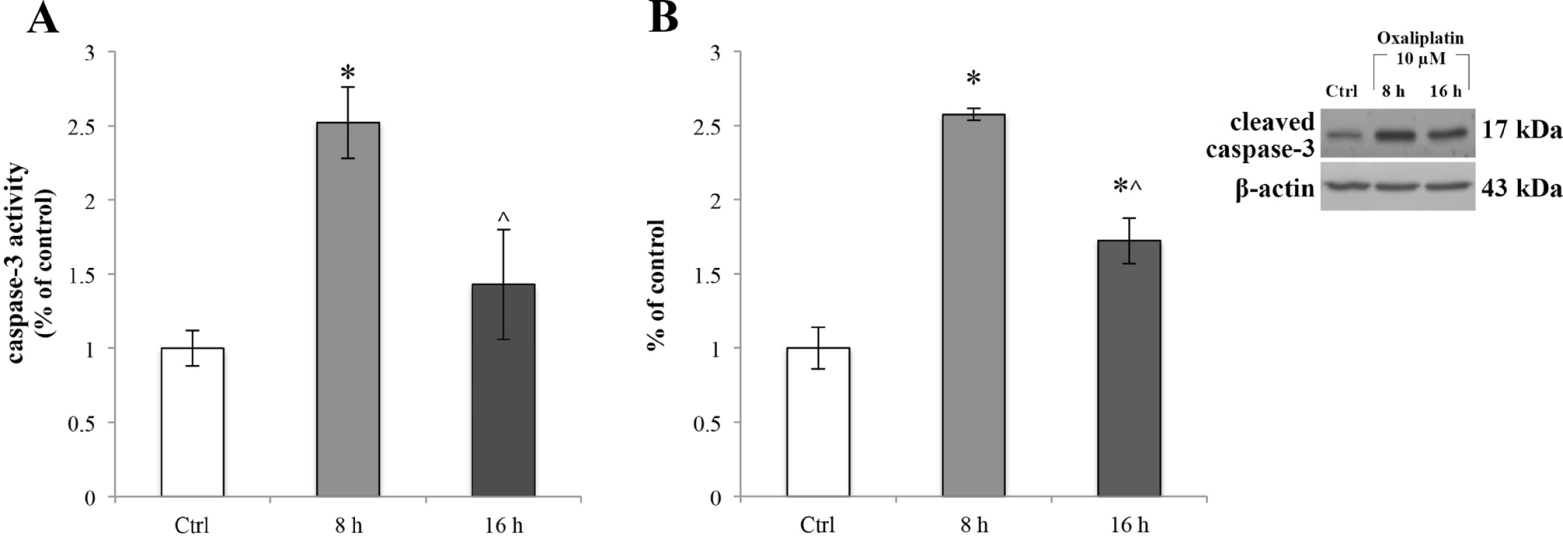
Caspase-3 activity in RBE4 cell after oxaliplatin treatment (**A**) Caspase-3 activity, revealed by fluorogenic peptide-based assay and (**B**). upregulation of caspase-3 cleavage, revealed by Western blot assay in RBE4 cells, post 10 μM oxaliplatin treatment for 8 and 16 h. β-actin was used as an internal control. Control condition was arbitrarily set as 100 % and results are expressed as mean ± S.E.M. All oxaliplatin treatment experiments were conducted independently in triplicate. Significant at ^*^p < 0.05 vs control and ^*p* < 0.05 vs 8 h.

Considering that ER stress can induce the activation of caspase-3 [39], we checked the activation of this downstream signal of ER stress evaluating the caspase-3 enzymatic activity (Figure 6A) in the oxaliplatin-treated RBE4 cells using the EnzChek^®^ Caspase-3 Assay Kit. We found that oxaliplatin significantly (^*^p < 0.05 vs control) activated the caspase 3 at 8 h of treatment (Figure 6A, light grey bar). Also, oxaliplatin-activated caspase-3 reaction was confirmed by Western blot assay (Figure 6B, light grey bar) showing an upregulation of the cleaved form of the protease. Moreover, in Figure 6A the result showed a time-dependent increase of caspase-3 activity that at 16 h of oxaliplatin treatment returned to control levels (^*p <* 0.05 vs 8 h) (Figure 6A, dark grey bar).

### Oxaliplatin-dependent extracellular ATP release

**Figure 7:**
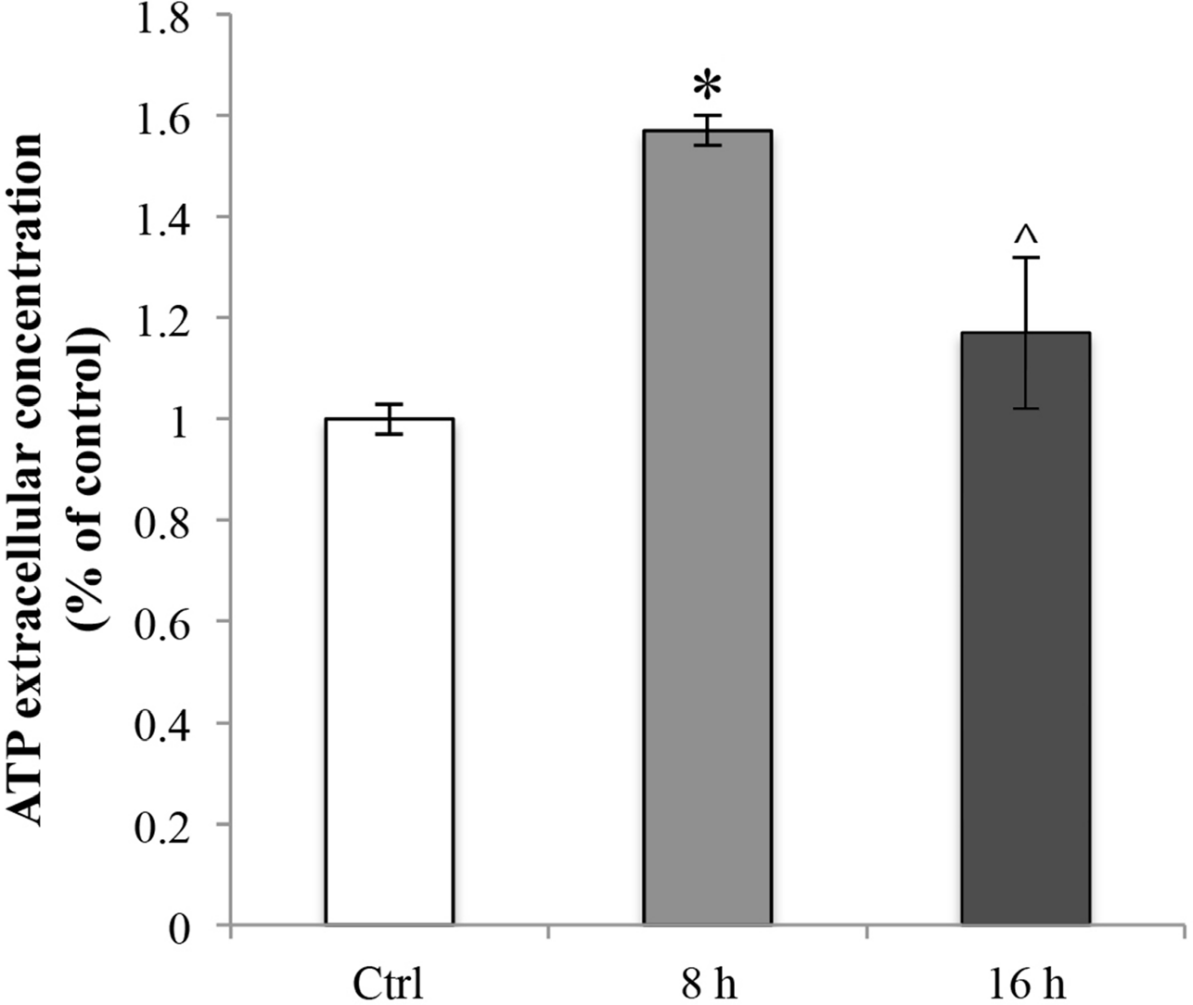
Oxaliplatin-dependent release of ATP Extracellular ATP release from RBE4 cells in the presence of 10 μM oxaliplatin. Control condition was arbitrarily set as 100% and results are expressed as mean ± S.E.M. *n* = 3, ^*^p < 0.05 vs control; ^*p* < 0.05 vs 8 h. All oxaliplatin treatment experiments were conducted independently in triplicate.

To characterize if RBE4 cells extrude ATP in response to oxaliplatin and eventually the mechanisms through which it occurs, firstly we evaluated the extracellular ATP release in RBE4 cells that underwent 10 μM oxaliplatin treatment. In cultures exposed for 8 h to oxaliplatin, baseline ATP release was significantly (^*^p < 0.05 vs control) elevated as compared with control cells (Figure 7, light grey bar). The phenomenon was transient since after 16 h of treatment with the chemotherapy agent the ATP levels returned to values comparable to those of control cells (^*p <* 0.05 vs 8h) (Figure 7, dark grey bars).

### Oxaliplatin-dependent ATP release is PANX1 independent

**Figure 8:**
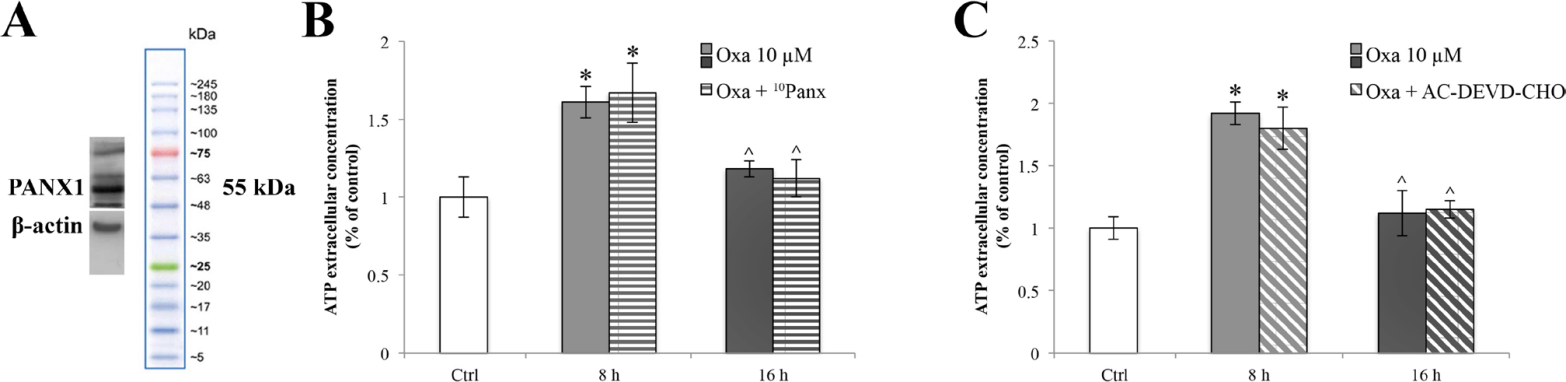
PANX1 channels expression in RBE4 cells and release of ATP during oxaliplatin treatment with ^10^Panx and AC-DEVD-CHO Western blot densitometry analysis of PANX1 protein expression levels in RBE4 cells (**A**). ATP quantification assay performed in the presence of 100 μM of ^10^Panx (**B**), or 100 μM AC-DEVD-CHO (**C**) demonstrating that both PANX1 and caspase-3 inhibitors did not affect the ATP release. Assays were performed in triplicate. Data are expressed as mean ± S.E.M. Data are representative of three independent experiments. ^*^p < 0.05 vs control; ^*p* < 0.05 vs 8 h.

In order to verify if ATP release is dependent on pannexin-1 (PANX1) activation, we decided to examine whether RBE4 cells express PANX1 by immunoblot analysis. Figure 8A shows that RBE4 cells clearly expressed anti-PANX1 (~55 kDa) immunoreactivity in relatively high density as compared to β-actin standard protein levels.

The role for PANX1 in ATP release in RBE4 cells was then examined. RBE4 cells were treated with oxaliplatin for 8 and 16 h, and ATP extrusion was measured in the presence and absence of the selective PANX1 inhibitor ^10^Panx. We observed that the pretreatment with ^10^Panx did not result in significantly blunt oxaliplatin-dependent ATP release (Figure 8B).

Finally we assessed whether caspase-3 induced PANX1 opening in RBE4 cells by evaluating ATP release after treatment with the AC-DEVD-CHO caspase-3 inhibitor. Results (Figure 8C) showed that the application of AC-DEVD-CHO did not attenuate the ATP extrusion.

Since neither the pharmacological inhibition of PANX1 nor the caspase-3 inhibition did not prevent the ATP spillage, we interpreted these data as showing that PANX1-mediated ATP release was not responsible for the ATP extrusion.

## DISCUSSION

To study the mechanisms involved in the generation of oxaliplatin neurotoxicity, we have explored the specific cellular and molecular processes underlying the blood brain barrier endothelial cells events that occur in the presence of the anticancer drug. Using a rat brain endothelial cell line (RBE4 cells), we demonstrated that oxaliplatin administration caused significant changes in the junctional and cytoskeletal apparatus of endothelial cells, thus reducing their tightness. These oxaliplatin-dependent alteration of the BBB could be, at least in part, associated with higher distribution of molecules that cross a defective BBB, and act in the CNS cooperating in pain chronicization.

Our data are in accordance with reported evidence that BBB, BSCB (Blood Spinal Cord Barrier), and BNB (Blood Nerve Barrier) disruptions participate in the complex mechanisms that initiate or maintain neuropathic pain [40–43].

Data concerning the mechanism of endothelial cellular uptake of oxaliplatin that is strictly dependent on the OCT2 *(Organic Cation Transporter)* [34], as well as the presence of these channels on the RBE4 plasma membrane [33] let us to hypothesized a direct interaction between oxaliplatin and BBB endothelial cells. Once inside the cells, oxaliplatin triggers an intracellular signaling pathway that results in a transient and reversible alteration (a sort of loosening) of the barrier itself.

Special characteristics of RBE4 cells include the presence of tight junctions. Structurally, the inter-endothelial tight junction complexes comprising the membrane proteins occludin and claudins and membrane-directed scaffolding proteins, such as ZO-1, contribute to the physical barrier nature of BBB and strictly limit the molecular/cellular influx from circulation [44]. Moreover, ZO-1 serves to anchor tight junctions (TJs) to the actin cytoskeleton in the cell [45–47] and F-actin has a critical role in establishing inter-endothelial junctional integrity and defining the peripheral morphological belt of endothelial cells [48, 49]. The re-distribution of major TJ proteins can result in reduced endothelial barrier tightness [50]. We evaluated the TJ protein ZO-1 and the cytoskeletal F-actin in order to evidence oxaliplatin-induced physiopathologic alteration. As a result, we found that oxaliplatin induced the dislocation of ZO-1 and the formation of F-actin stress fibers.

Previous studies have shown the important role of oxidative stress in the toxicity of oxaliplatin [11, 37, 51–53]. An excessive production of ROS can alter endothelial cells integrity by cytoskeletal rearrangements and redistribution of TJ proteins, triggering a loss of cellular interaction and increasing their permeability [54–57]. We therefore hypothesized that oxaliplatin, once entered the endothelial cells by OCT2 might have an effect on ROS production, which could possibly be accompanied by TJ dislocation. In the present study, it has shown that oxaliplatin induced a time-dependent increase in ROS production, indicating that oxaliplatin-dependent oxidative stress could be an important signal involved in the disruption of the integrity of the endothelial junctional apparatus.

In response to oxidative stress mediated by ROS, accumulation of unfolded or misfolded proteins triggers the cellular adaptive procedure known as ER stress [58, 59]. By mediating the shutdown of general protein synthesis and increasing the production of molecular chaperones, including glucose-regulated protein 78 (GRP78), ER stress is designed to be protective [60–63]. Recently, ER stress has been shown to play a detrimental role on TJs integrity [64]. In fact, ER stress can also accelerate ROS production both in the ER and mitochondria [65–67], that in turn activate apoptotic signaling pathway [68, 69]. Considering that in our condition the mitochondria-dependent pro-apoptotic protein BAX was not activated, we decided to investigate the changes in GRP78 expression levels. Our findings showed that GRP78 is induced in a time-dependent manner, suggesting that ER stress is activated at 8 h after oxaliplatin treatment and that ROS could be the initiator of the ER stress.

It is well known [63, 64, 66–70] that the ER stress response is a mechanism that the cell puts in place in order to react to perturbations in ER homeostasis, during which GRP78 functions as a cytoprotective protein. Thus, the oxaliplatin-dependent upregulation of GRP78 could be to relieve the ER stress and restore cell function. It has been also suggested that the caspase-3 activation is one of the mechanisms that operate such protection, recognizing and removing overproduced ROS [71–74]. According to these data, our results showed an oxaliplatin-dependent activation of caspase-3 in absence of a parallel activation of the apoptotic signalling cascade (demonstrated by the lack of an increase in BAX protein expression levels). On the other hand, other authors have demonstrated that caspase-3, cleaving the PANX1 autoinhibitory C-terminal domain, induces the functional activation of PANX1 [75], a channel known to have a prominent role in the release of ATP [53, 76]. Indeed, recent findings revealed that chemotherapeutic drugs induce ATP release [77] that, in turn, leads to degradation of tight junctions, such as ZO-1 [78]. Furthermore, Boyd-Tressler and co-authors have fully demonstrated that diverse chemotherapy agents, induce functional activation of PANX1 [10, 79]. Driven by these results, we hypothesized that oxaliplatin-dependent caspase-3 activation could elicit an ATP spillage by PANX1 channels. To our knowledge this work demonstrates for the first time that RBE4 cells express PANX1 channels, confirming previous data showing that these cells are a useful *in vitro* model to study the BBB [32, 80]. Moreover, we demonstrated that oxaliplatin induces an ATP release and that this leakage is independent of the PANX1 opening. This result is not surprising since the caspase-3-mediated mechanism of PANX1 activation is closely related to the onset of the apoptotic pathway [81], a condition that does not occur in our experimental condition.

Moreover, this data agrees with the maintenance of ATP release in the presence of a caspase-3 inhibition. A possible, early, homeostatic signaling mediated by caspase-3 activation is suggested. By contrast, the cellular mediator able to evoke the release of the excitotoxic molecule ATP remains unclear deserving further research.

In summary, our data provide evidence of a chemotherapy-dependent BBB alterations that, starting from ROS formation, activates the ER stress/ATP release signalling pathway in RBE4 cells. We have conclusively shown that activation of this pathway, causes alterations of the TJ protein ZO-1 and of the cytoskeletal protein F-actin. These findings contribute to identify the signal pathways of chemotherapy-induced opening of the BBB and emphasizes the importance of dissecting in detail the molecular mechanism that alters the BBB during chemotherapy treatment in order to establish potential therapeutic targets in the treatment of chronic pain management.

## MATERIALS AND METHODS

### Cell culture and treatments

The rat brain endothelial cell line RBE4 was kindly provided by Dr Vincenzo Giuseppe Nicoletti (Department of Biomedical Sciences, University of Catania, Italy). Cells (up to passage 20) were grown and maintained in alpha-minimal essential medium (alpha-MEM)/Ham's F10 (1:1) (GIBCO, Thermo Fisher Scientific, Milan, Italy) supplemented with 10% fetal bovine serum (FBS), 1% penicillin/streptomycin, 2 mM L-glutamine (EuroClone, Milano, Italy) and 1 ng/ml basic fibroblast growth factor (bFGF) (Thermo Fisher Scientific, Milano, Italy) at 37° C, 5% CO_2_ in humidified atmosphere. The growth medium was routinely changed 2–3 times per week. For each experimental set-up, the cells were seeded on appropriate support for 24 h in complete growth medium. The day of the stimulation, the complete growth medium was replaced with starvation medium (without FBS and without bFGF) containing appropriate stimuli at different concentration.

^10^Panx (Proteogenix, Schiltigheim, France) was dissolved in 1% DMSO (dimethyl sulfoxide) to obtain 1 mM solution and then diluted in physiological medium at final concentration of 100 μM in the presence of oxaliplatin for 8 or 16 h. This concentration was chosen accordingly with previous published data [10, 82, 83]. The caspase-3 inhibitor AC-DEVD-CHO (N-Ac-Asp-Glu-Val-Asp-CHO) (Calbiochem, Milan, Italy) was incubated for 2 hours at a concentration of 100 μM before oxaliplatin treatment. A non-toxic effect of AC-DEVD-CHO at this concentration has been demonstrated previously [65].

### MTT assay

Cell viability was evaluated by the reduction of 3-(4,5-di-methylthiozol-2-yl)-2,5-diphenyltetrazolium bromide (MTT) by mitochondrial dehydrogenase, that directly reflects the activity of mitochondria, and can be considered an indirect measurement of cell viability. RBE4 cells were plated into 96 multiwell plates, at the density of 2.5 × 10^4^ cells/well, in their appropriate complete growth medium. The following day, the cells were starved as described above and treated with different concentrations of oxaliplatin (1–3–10–30–100 μM), for 8, 16 and 24 h. After treatment, the starvation medium containing stimuli was removed and substituted with 1 mg/mL MTT (in starvation medium without phenol red). The chromogenic solution was incubated for at least 20 min. at 37° C. The formazan crystals formed were dissolved by adding 100 μl of DMSO in each well and the absorption at 570 nm was read using a microplate reader (MultiskanFC^™^ microplate photometer, ThermoFisher Scientific, Milan, Italy). Each experimental point was performed in quintuplicate, for three times.

### Western blotting

RBE4 cells, at the density of 3.5 × 10^6^ cells, were plated in Petri dishes in their appropriate complete growth medium. After 24 h the cells were serum starved and stimulated with oxaliplatin 10–30 μM, for 8, 16, and 24 h. After treatments, the medium was removed and cells were scraped in cold PBS (Phosphate Buffer Saline). The cell suspensions were centrifuged at room temperature (RT) for 10 min. at 1000 rpm. The pellets obtained were treated with Mem-PER^™^ Plus Membrane Protein Extraction Kit (Thermo Fisher Scientific, Milan, Italy) following the manufacture instruction, to isolate the integral and attached membrane proteins isolated from the cytosolic proteins. The protein concentration was evaluated by the Bradford method and equal amounts of protein (15 μg) were separated on a 12% polyacrylamide gel by electrophoreses and transferred onto nitrocellulose membrane (Porablot NPC, MACHEREY-NAGEL, Milan, Italy). After 1 h blocking with 3% bovine serum albumin (BSA) in Tris-buffered saline containing 0.1% Tween 20 (Tween-TBS) at RT, the blots were incubated in Tween-TBS/3% BSA overnight at 4° C with the following primary antibodies; rabbit primary antibody for 78 kDa glucose-regulated protein (GRP78) (ThermoFisher Scientific, Milan, Italy), for cleaved caspase-3 (Cell Signalling, Euroclone, Milan, Italy), for BCL2 associated X protein (BAX) (Santa Cruz Biotechnology, Milan, Italy), and goat primary antibody for (PANX1) (Santa Cruz Biotechnology) at 1:500, 1:1000 and 1:300, and 1:500, respectively. After washing with Tween-TBS, the goat anti-rabbit HRP secondary antibodies (Santa Cruz Biotechnology) was added at 1:5,000 dilution in Tween-TBS for 1 h at room temperature (RT), and washed again. Proteins were detected with the Amersham ECL Plus Western Blotting Detection Reagent (GE Healthcare, Milan, Italy). Protein expression levels were then quantified by the ImageJ analysis software (ImageJ, National Institute of Health, USA, http://imagej.nih.gov/ij). β-actin (1:10000 dilution) (Santa Cruz Biotechnology) normalization was performed for each sample. Each experiment was performed three times, in triplicate.

### ZO-1 tight junction and F-actin immunofluorescent labeling

RBE4 cells were seeded on sterilized cover slips (lodged in a 6 multiwell plate) in the number of 1.5 × 10^5^ cells, in their appropriate complete growth medium. After 24 h the cells were serum starved and stimulated with oxaliplatin 10–30 μM, for 8, and 16 h. After treatments, the medium was removed and each cover slip was washed twice with cold PBS followed by fixation with paraformaldehyde 3.7% (for F-actin) in PBS for 10 min. at room temperature or cold methanol (for Zonula Occludens-1, ZO-1) for 20 min. After fixation, two more washes with cold PBS were performed, the cover slips were dried at RT for at least 1 h. The fixed cells where rinsed in cold PBS for three time and permeabilized with a 0.1% solution of TRITON X-100 in PBS for 10 min at RT. Following, cells were washed three times with PBS and then incubated for 15 min. in a blocking solution (1% BSA in PBS) at RT. Each cover slip was incubated overnight at 4° C with the rabbit primary antibody anti-ZO1 (1:50) or with Alexa-488-conjugated phalloidin (F-actin) (1:200; ThermoFisher Scientific, Milan, Italy), in 1% BSA. The day after, cells were washed three times with PBS and each cover slip was incubated in goat anti-rabbit (for ZO-1) immunoglobulin G (IgG) secondary antibody, conjugated with Alexa Fluor 568 (1:200; Invitrogen, Milan, Italy) for 1 h at RT. Then (for ZO-1 and F-actin) after counterstaining with DAPI (4’,6-diamidin-2-fenilindolo; 1:2000 dilution; Invitrogen, Milan, Italy) for 5 min at RT, cover slip glasses were mounted using Fluoromount anti-fade solution (ThermoFisher Scientific, Milan, Italy) on cover slides. Fluorescent signals were detected at 400× total magnification (five microscopic fields for each experimental point) by a motorized Leica DM6000B microscope equipped with a DFC350FX camera. Negative controls were performed by omitting the primary antibody to confirm the specificity of primary antibodies used and by omitting the second antibody to reveal autofluorescent labeling (data not shown). Fifteen pictures from each field were captured. The data shown represent the typical data from three independent experiments that yielded similar results. Each experiment was performed in triplicate.

### Quantification of intracellular ROS by flow cytometry

RBE4 cells were plated in Petri dishes in their appropriate complete growth medium, reaching about 80% of confluence. After that, the cells were starved as reported above and stimulated with 10 μM of oxaliplatin, for 1, 2, 4, 6 and 8 h. After each treatments, cells where washed twice with DMEM w/o phenol red and detached from Petri dishes surface by trypsin/EDTA and centrifuged at 1000 rpm for 5 min. at room temperature. The pellets were gently resupended in DMEM w/o phenol red and labelled with 1 μM CM-H_2_DCFDA (Life Technologies, ThermoFisher Scientific, Milan, Italy). The tubes were gently mixed and dark incubated at 37° C for 20 min. After labelling, cells were centrifuged again, the supernatant discard and the pellets obtained were gently resuspended in DMEM w/o phenol red, and immediately analyzed using a FACSCanto flow cytometer (Becton–Dickinson, San Jose, CA, USA).

The sample flow rate was adjusted to about 10^3^ cells/s. For a single analysis, the fluorescence properties of about 2.5 × 10^4^ RBE4 cells were collected. Each experiment was performed three times, in triplicate.

### Caspase-3 enzimatic activity

RBE4 cells were plated in 6-well plates (5 × 10^5^/well) in appropriate complete growth medium. The following day, cells were starved and stimultated with oxaliplatin 10–30 μM for 8 and 16 h. After treatment, cells were scraped in 100 μl lysis buffer (200 mM Tris–HCl buffer, pH 7.5, containing 2 M NaCl, 20 mM EDTA, and 0.2% Triton X-100). Fifty μl of the supernatants were incubated with 25 μM fluorogenic peptide caspases 3 substrate rhodamine 110 bis-(N-CBZ-L-aspartyl-L-glutamyl-L-va- lyl-L-aspartic acid amide) (AC-DEVD-CHO; Molecular Probes) at 25° C for 30 min. The amount of cleaved substrate in each sample was measured in a 96-well plate fluorescent spectrometer (Perkin-Elmer; excitation at 496 nm and emission at 520 nm). Each experiment was performed three times, in triplicate.

### Extracellular ATP quantification

RBE4 cells were plated in 6-well plates (5 × 10^5^/well) in appropriate complete growth medium. The following day, cells were starved and stimultated with oxaliplatin 10–30 μM for 8 and 16 h. After treatment, the medium was harvested and 50 μl were processed following the manufacture's procedure (ATPlite – Luminiscence ATP Detection Assay System, PerkinElmer Italia, Milan, Italy). The extracellular ATP was quantified by luminescence using a VICTOR microplate reader (PerkinElmer, Milan, Italy). Each experiment was performed three times, in triplicate.

### Statistical analysis

Statistical analyses were performed by Two-way ANOVA followed by the Mann–Whitney test. All assessments were made by researchers blinded to treatments. Data were analysed using “Origin 9” software (OriginLab, Northampton, USA). Differences were considered significant at *p <* 0.05.
